# How legal problems are conceptualized and measured in healthcare settings: a systematic review

**DOI:** 10.1186/s40352-023-00246-5

**Published:** 2023-11-18

**Authors:** Joshua R. Vest, Rachel J. Hinrichs, Heidi Hosler

**Affiliations:** 1 Indiana University Richard M Fairbanks School of Public Health, Indianapolis, IN USA; 2 Center for Biomedical Informatics, Regenstrief Institute, Indianapolis, IN USA; 3 University Library, Indiana University Purdue University Indianapolis, Indianapolis, IN USA

**Keywords:** Social determinants of health, Screening, Measurement

## Abstract

Legal problems encompass issues requiring resolution through the justice system. This social risk factor creates barriers in accessing services and increases risk of poor health outcomes. A systematic review of the peer-reviewed English-language health literature following the PRISMA guidelines sought to answer the question, how has the concept of patients’ “legal problems” been operationalized in healthcare settings? Eligible articles reported the measurement or screening of individuals for legal problems in a United States healthcare or clinical setting. We abstracted the prevalence of legal problems, characteristics of the sampled population, and which concepts were included. 58 studies reported a total of 82 different measurements of legal problems. 56.8% of measures reflected a single concept (e.g., incarcerated only). The rest of the measures reflected two or more concepts within a single reported measure (e.g., incarcerations and arrests). Among all measures, the concept of incarceration or being imprisoned appeared the most frequently (57%). The mean of the reported legal problems was 26%. The literature indicates that legal concepts, however operationalized, are very common among patients. The variation in measurement definitions and approaches indicates the potential difficulties for organizations seeking to address these challenges.

**Supplementary Information:**

The online version contains supplementary material available at 10.1186/s40352-023-00246-5.

## Introduction

Patients with past and current legal problems face significant barriers in accessing key services and are at risk of future poor health outcomes and high cost services. In the US, more than 3.8 million adults are on probation or parole (Kaeble, 2020), which are associated with increased emergency department utilization and hospitalizations (Hawks et al. 2020). Additionally, the US has the highest incarceration rates in the world (The Sentencing Project, 2022) and patients with a history of incarceration are at increased risk for chronic conditions and face barriers to housing and employment (Massoglia & Pridemore, 2015). As part of the broader trend in social risk factor measurement, healthcare organizations are able to screen for patients’ legal problems through the wide variety of questionnaires developed by healthcare organizations, government agencies, practice collaboratives, and electronic health record vendors (Social Interventions Research Evaluation Network. Social Needs Screening Tool Comparison Table, 2019).

However, substantial variation appears to exist in the how healthcare organizations have operationalized patients’ legal problems. For example, screening questionnaires and surveys use differing words, including time in jail, parole, arrests, justice-involvement, and incarceration (Moen et al. 2020; Saloner et al. 2016; Social Interventions Research Evaluation Network. Social Needs Screening Tool Comparison Table, 2019), to capture patients’ legal problems. Likewise, the interventions available to healthcare organizations to support patients, such as medical-legal partnerships, may address differing legal problems such as issues arising from criminal records, immigration status, and broader issues such as housing and benefits (Sandel et al. 2010). Also, ICD10 Z codes allow for problems related to arrests and prosecution in addition to incarceration history (World Health Organization. ICD-10 Version:, 2019. Chapter XXI Factors influencing health status & contact with health services 2019).

The objective of this study is to answer the question, how has the concept of patients’ “legal problems” been operationalized in healthcare settings? Given that healthcare organizations are increasingly attentive to identifying patients’ social risk factors, we specifically focus on the operationalization of “legal problems” in the screening and measurement contexts. Clarity on the difference between definitions of patients’ legal problems in current measurements will allow organizations to link services more effectively to patients’ needs and enable comparisons across populations.

## Theoretical framing

The objectives and orientation of this review are grounded in the epidemiological concept of screening and the population health management concept of risk stratification. Screening for social risk factors, such as patients with legal problems, is simply a systematic process of case finding (Andermann, 2018). Risk stratification is the process of subdividing a large population into smaller segments at increased risk for negative health outcomes (Girwar et al. 2021). The identification of key subsets within the population allows for better matching of needed resources to specific patient needs through referrals or direct service provision (Vuik et al. 2016). In population health management, social risk factor screening may be used to inform, or be the basis of, risk stratification and intervention delivery (Steenkamer et al. 2017).

To be effective, however, case finding via screening and stratification strategies must accurately reflect the social factor of concern. Incorrect identification of a patient’s specific risk could result in services that are poorly matched or altogether neglect patient needs. The former is a potential waste of resources, and the latter does not improve patient health. For example, common screening questionnaires’ language reflects concepts such as time in jail, parole, arrests, justice-involvement, and incarceration (Moen et al. 2020; Saloner et al. 2016; Social Interventions Research Evaluation Network. Social Needs Screening Tool Comparison Table, 2019). While each of these different terms are potentially reflective of the broader concept of legal problems (Currie, 2009), these terms have specific definitions that reflect different types of engagement with the criminal justice system, differ in terms of temporality, and may have different risks for health (Bryson et al. 2021). For example, individuals with a history of arrests may face stigma and discrimination while seeking care from the health care system (Redmond et al. 2020; Smith et al. 2022). During incarceration, risks include infectious disease, violence, and substance misuse (American Academy of Family Physicians, 2021). Individuals recently released from incarceration can face difficulties in employment or housing, which affects access to the resources and environments to remain healthy (Lares & Montgomery, 2020).

## Methods

We conducted a systematic review of the peer-reviewed English-language health literature following the PRISMA guidelines (Page et al. 2021). We included the following concepts under the broader term legal problems: probation, arrest, parole, incarceration, criminal record/history, corrections, justice-involved and juvenile justice. Known, relevant articles collected by the authors were analyzed to select relevant keywords and subject headings. We excluded legal problems of a civil nature (i.e. divorce, custody, lawsuits, etc.).

### Information sources & search strategy

We identified peer-reviewed articles through database searches. First, the teams’ health science librarian (RH) queried Medline (via OVID), PsycINFO (via EBSCO), CINAHL (via EBSCO), the Criminal Justice Index (via ProQuest), and Google Scholar on March 9^th^, 2022. The first 100 records were downloaded from Google Scholar. The final search terms incorporated subject headings and keywords associated with legal problems, social determinants of health, screening and instruments, and healthcare settings.

Articles were eligible for inclusion if they reported the measurement or screening of individual patients for legal problems in a US healthcare or clinical setting. We excluded all nonpatient populations and settings, such as assessments of currently incarcerated populations, studies of clinician perceptions, studies within community-based organizations, or national population-based surveys. We excluded all articles that were case studies, commentaries, or editorials, and those not in English. The full search strategies for all information sources are provided in Additional file 1.

### Selection and screening

First, two authors (JV and HH) independently screened the titles for potential inclusion. The goal at this step was to exclude the obviously ineligible studies. As such, we erred on the side of inclusion. The second round was screening based off the abstract information only. From this set, we reviewed the full text to determine final inclusion status. The interrater agreement on this final step was 0.65. All differences were resolved with a joint reading and discussion until consensus was reached.

### Data elements and abstraction

One author (HH) independently abstracted data, which was verified by a second author (JV). The data abstracted included identifying information from each of the included studies: author(s), year, title, and journal. In addition, the study design, study dates, healthcare setting, method of patient data collection (e.g., survey, natural language processing (NLP), review of data charted by providers), and study sample demographics were abstracted.

Next, we extracted each article’s reported measures of legal problems. One article may have had more than one measure. For each of the reported measurements, we used a series of binary indicators to indicate which concepts were included in the measure: incarceration, jail, probation, parole, arrests, awaiting trial, contact with criminal justice system, convictions, crimes, immigration status, or juvenile detention. If provided, we also abstracted the reported prevalence of legal problems. We noted if any measurement of the reliability and validity was reported.

### Bias assessment

To understand the potential bias in the reported measures of legal problems, we described each study sample broadly as general patient populations, or targeted to those with specific risk behaviors or characteristics (i.e. at-risk).

### Analyses

We describe the characteristics of the included studies and provided measures using frequencies and percentages. Additionally, we used means to summarize the reported percentages of legal problem by including concepts stratified by general or at-risk populations. We compared means using t-tests. Due to small samples, we limited the summarization by means to those categories with at least 10 observations.

## Results

### Study selection

**Fig. 1:**
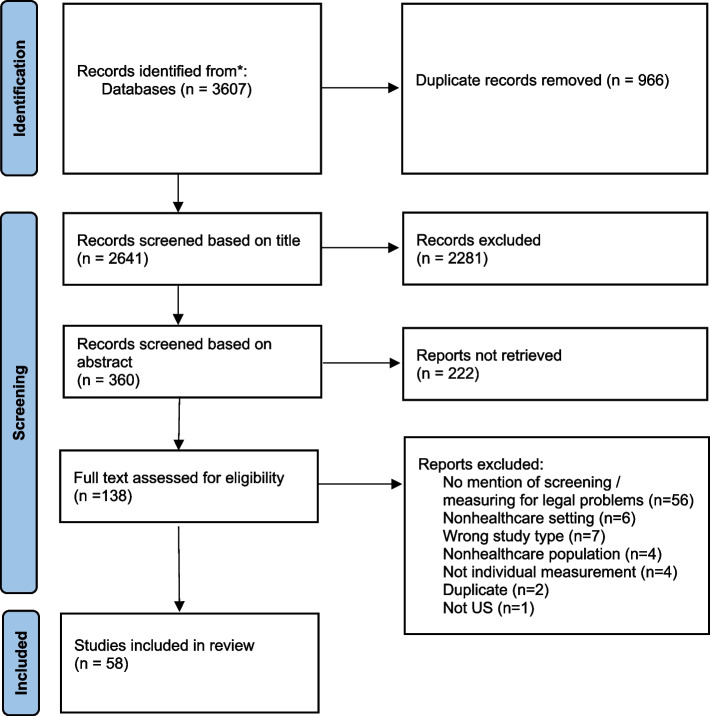
Search strategy to identify healthcare's operationalization of legal problems

Our initial search strategy yielded 2,641 records for screening after duplicates were removed (Fig. 1). After excluding records based on title (*n* = 2,281) and abstract (*n* = 222), we were left with 138 records for full-text assessment. The most common reason for excluding the study at this stage was no mention of screening or measurement of legal problems (*n* = 58). The other reasons for exclusion (e.g. non-healthcare settings or non-healthcare population) were much less frequent. The resultant strategy and selection process resulted in 58 studies that reported a total of 82 different measurements of legal problems (Additional file 2).

### Study characteristics

The included studies utilized patient samples from a variety of healthcare delivery settings (Table 1). Notably, a fifth of the studies were focused on behavioral health settings. Other settings included specialty providers such as STD (Weisbord et al. 2003; Widman et al. 2014) or methadone clinics (Magura et al. 1998). The patients represented in the included studies were largely adults (75.4%) and inclusive of both genders (92.6%). However, several of the studies, notably those among veterans, were highly skewed towards male samples (Blosnich et al. 2020; Elbogen et al. 2018; Holliday et al. 2021; Schultz et al. 2015; Szymkowiak et al. 2022; Wang et al. 2019). Overall, the included studies focused more on patient groups with some known risk factors associated with legal problems (61.4%), such as behavioral health comorbidities (Anderson et al. 2015; Buzi et al. 2016; Carlsen-Landy et al. 2020; Evens & Vander, 1997; Giggie et al. 2007; Harry & Steadman, 1988; Klassen & O’Connor, 1988; Lorine et al. 2015; Pasic et al. 2005; Phillips et al. 2002; Prins et al. 2015; Rich & Sullivan, 2001; Schauss et al. 2020; Theriot & Segal, 2005), history of risky sexual behaviors (Aronson et al. 2016; Kadivar et al. 2006; Sheu et al. 2002; Tolou-Shams et al. 2007; Widman et al. 2014), or at risk of substance misuse (Claus & Kindleberger, 2002; Liebschutz et al. 2010; Magura et al. 1998; Mark et al. 2020; Pittsenbarger et al. 2017; Schultz et al. 2015; Wang et al. 2010).

**Table 1:** Measures of legal problems in healthcare and study characteristics

Characteristic	Percent
Setting^a^	
Behavioral health	21.1
FQHC	5.3
Health system (inpatient & outpatient)	29.8
Hospital or emergency department	19.3
Primary care clinic	14.0
Other clinic type	10.5
Population^a^	
General	38.6
At risk	61.4
Age groups included^a^	
Adults	75.4
Pediatric	14.0
Both	10.5
Genders included^a^	
Both	92.6
Female only	4.4
Male only	2.9
Measurement method^b^	
Survey	63.4
Linked databases	14.6
Chart review	9.8
EHR structured data	8.5
NLP	3.7
Number of concepts appearing within measures^b^	
One	56.8
Two	21.0
Three	13.6
Four	2.5
Five	6.2
Concepts appearing within measures^b^	
Incarceration	58.5
Arrests	26.8
Jail	25.6
General / no description contact with criminal justice system	13.4
Juvenile detention / correction	13.4
Convictions	12.2
Parole	11.0
Probation	11.0
Immigration	6.1
Criminal activity	1.2

^a^Out of articles (*n* = 58)

^b^Out of individual measures (*n* = 82)

The most common method for obtaining individual’s legal problems (Table 1) was through a survey or questionnaire (63.4%). Most of these instances were homegrown or unique tools and only a few studies reported using previously published multidomain social determinant of health screeners like PRAPARE (Kusnoor et al. 2018) or I-HELLP (Ko et al. 2016; Patel et al. 2018). However, multiple studies made use of the legal module within the Addiction Severity Index (ASI) (Claus & Kindleberger, 2002; Erlyana et al. 2019; Schultz et al. 2015; Wang et al. 2010), which is a psychometrically evaluated tool for measuring substance abuse behavior and related risk factors. The next most common approach to measuring legal problems was through the use of linked databases (14.6%), i.e., the combination of data sources created and maintained by disparate entities through patient identifiers (Arthur et al. 2018; Claassen et al. 2007; Evens & Vander, 1997; Finlay et al. 2022; Harry & Steadman, 1988; Klassen & O’Connor, 1988; Mark et al. 2020; Theriot & Segal, 2005). While not a true gold standard, these linked sources represent an independent and objective measurement of patient contact with aspects of the criminal justice system. The remaining approaches to measurement all relied on existing information from medical records. Beyond general “chart review”, several measures used structured data from within the electronic health record (EHR): ICD-10 Z codes (Alemi et al. 2020; Blosnich et al. 2020; Davis et al. 2020), specific service codes (Davis et al. 2020; Szymkowiak et al. 2022), or sources of admissions and discharges (Garrett et al. 2020). Lastly, three studies used NLP techniques to identify legal problems (Boch et al. 2021; Vest et al. 2017; Wang et al. 2019). Notably, Wang and colleagues (Wang et al. 2019) compared their NLP algorithm against an independent reference standard.

### Measures of “legal problems” used within individual studies

The included studies used a wide variety of terms to describe the constructs under consideration ranging from variants of global terms like criminal justice involvement, interaction, or status (Alemi et al. 2020; Anderson et al. 2015; Doran et al. 2021; Holliday et al. 2021; Ko et al. 2016; Magura et al. 1998; Ragucci et al. 2001; Schauss et al. 2020; Schultz et al. 2015; Shah et al. 2009; Theriot & Segal, 2005), correctional involvement (Boch et al. 2021; MacKenzie et al. 2021), criminal history (Carlsen-Landy et al. 2020; Claassen et al. 2007; Schultz et al. 2015), and legal problems or needs (Aronson et al. 2016; Blosnich et al. 2020; Davis et al. 2020; Heller et al. 2020; Kulie et al. 2021; Poleshuck et al. 2020; Tolou-Shams et al. 2007) to the more focused such as incarceration (Anderson et al. 2015; Buzi et al. 2016; Doran et al. 2021; Elbogen et al. 2018; Garrett et al. 2020; Gilbert et al. 2013; Howell et al. 2016; Lorine et al. 2015; MacKenzie et al. 2021; Mark et al. 2020; Pasic et al. 2005; Rich & Sullivan, 2001; Shah et al. 2009; Sheu et al. 2002; Szymkowiak et al. 2022; Wang et al. 2010; Widman et al. 2014), arrests (Doran et al. 2021; Erlyana et al. 2019; Harry & Steadman, 1988; Klassen & O’Connor, 1988; Rich & Sullivan, 2001), or immigration status (Gottlieb et al. 2014; Patel et al. 2018; Wyrick et al. 2017). Within this set, some articles provided very detailed definitions of the constructs being measured [e.g. (Gilbert et al. 2013; Schultz et al. 2015; Theriot & Segal, 2005), or provided a clear distinction between lifetime and more recent exposures [e.g. (Howell et al. 2016; Wang et al. 2019)].

Among the 82 different recorded measures (Table 1), just over half (56.8%) reflected a single concept (e.g., incarcerated only). The rest of the measures reflected two (21.0%) or more concepts within a single reported measure (e.g., incarcerations and arrests, or arrests, convictions, parole, probation, and incarceration). Among all measures, the concept of incarceration or being imprisoned appeared the most frequently (58.5%). The measures next most frequently reflected were arrests (26.8%) or time in jail (25.6%).

### Synthesis

Overall, the mean of the reported legal problems was 26.0% across all studies. When stratified by study population, the percent of legal problems among studies of general populations was 13.8%. This was statistically lower than the percentage among at risk populations (35.6%). This was the general trend for all the examined concepts. For example, when legal problems included the concept of incarcerations, the percent among at-risk populations was significantly higher (36.1%) than the percent among general populations (16.5%). Within population groups, the mean prevalence varied according to the included concepts. Regardless of measurement approach, those at risk generally had a higher percentage of legal problem than those measured among general populations (Table 2).

**Table 2:** Mean reported percentage of legal problems by concepts included in the definition and measurement methods

		At risk	General	
	n	Mean (SD)	Mean (SD)	p
Total^a^	77	35.6 (22.2)	13.8 (16.1)	0.0001
Concepts appearing within measures^b^				
Incarceration	45	36.1 (22.2)	16.5 (17.9)	0.0022
Arrests	21	41.9 (24.9)	20.3 (20.3)	0.0534
Jail	19	24.9 (20.4)	22.1 (19.7)	0.7930
General / no description contact with criminal justice system	9	26.6 (31.6)	2.4 (1.6)	0.1271
Juvenile detention / correction	10	41.6 (10.7)	18.7 (21.6)	0.0874
Convictions	9	40.6 (29.3)	2.1 (1.0)	0.0639
Parole / probation	9	34.0 (29.3)	16.0 (19.7)	0.3301
Immigration	5	34.0 (29.3)	16.0 (19.7)	0.3301
Measurement method^c^				
Survey	47	34.2 (22.5)	19.0 (16.5)	0.0131
Linked databases	12	45.3 (25.2)	2.2 (1.0)	0.0037
Chart review	8	35.6 (20.8)	–^c^	–
EHR structured data	7	21.3 (0.1)	2.5 (3.0)	0.0004
NLP	3	–	15.6 (23.5)	–

^a^Studies with no percentages reported omitted (*n* = 5)

^b^Concept appeared in definition, but not exclusively. More than one concept may be included

^c^Combination not present – not reported

## Discussion

Legal problems broadly encompass issues requiring resolution through the justice system (Currie, 2009; Nobleman, 2014). Legal problems, in their variety of manifestations, create barriers to health and wellbeing for patients. The existing literature on patients’ legal problems in healthcare settings utilize a variety of measurement methods and measures, including different and sometimes multiple concepts. Overall, the literature indicates that legal concepts, however operationalized, are very common among patient groups with known risk factors and common among general patient populations. The variation identified in measurement definitions, measurement approaches, and included populations indicates health care organizations will face challenges in formulating intervention strategies.

First, we focused on those problems associated with the criminal justice system. Even within this restricted definition of legal problems, we noted substantial variability in the concepts measured in the literature. This in itself is not a negative; different legal problems represent unique encounters with the criminal justice system and may create different risks or require different responses. For example, incarceration in prison is a detention due to a conviction (Gilbert et al. 2013) and that stress leads to negative health effects, which may require attentiveness to conditions such as heart disease or hypertension (Massoglia & Pridemore, 2015). In contrast, an arrest is a less severe degree of contact with the criminal justice system (Asad & Clair, 2018), but one that can create barriers to housing and employment (Ispa-Landa & Loeffler, 2016). However, the challenge with the literature was in frequent lack of specificity and clarity around measurement definitions without such clarity, matching appropriate interventions becomes more difficult.

Second, the validity and reliability of the measurement strategies selected was largely unknown; pragmatic tools or unspecified items or definitions were most common. In this respect, the operationalization of legal problems has the same challenges as other social risk factors, which tend to have poorly evaluated measurement tools (Henrikson et al. 2019). This practice of non-validated or programmatic tools unfortunately contributes to the uncertainty of the measurements. In addition to lack of clarity, some tools included multiple distinct concepts simultaneously. In contrast, those that relied on the Addiction Severity Index (ASI) could be much more specific about, and confident in, the reliability and validity of their measures of legal problems (Claus & Kindleberger, 2002; Erlyana et al. 2019; Schultz et al. 2015; Wang et al. 2010). However, using a validated tool like the ASI may not be practical in practice or in research studies given its length. For those looking to use various screening methods, the study by Wang and colleagues (Wang et al. 2019) was particularly useful as it indicated that measures were generally very specific, but were not as sensitive. Regardless of the survey or NLP algorithm selected, an effective process will require clear definition of the issue being measured so that the correct intervention can be identified and delivered.

As a result of these measurement issues, along with the frequent study of at-risk populations, makes drawing conclusions about the prevalence of legal issues in US healthcare setting challenging. At risk populations had much higher reported percentages of legal problems than general patient populations. Attempts to generalize these percentages to the general patient populations is likely not possible, as legal problems are associated with behavioral health comorbidities (Anderson et al. 2015; Buzi et al. 2016; Carlsen-Landy et al. 2020; Evens & Vander, 1997; Giggie et al. 2007; Harry & Steadman, 1988; Klassen & O’Connor, 1988; Lorine et al. 2015; Pasic et al. 2005; Phillips et al. 2002; Prins et al. 2015; Rich & Sullivan, 2001; Schauss et al. 2020; Theriot & Segal, 2005), history of risky sexual behaviors(Aronson et al. 2016; Kadivar et al. 2006; Sheu et al. 2002; Tolou-Shams et al. 2007; Widman et al. 2014), or at risk of substance misuse (Claus & Kindleberger, 2002; Liebschutz et al. 2010; Magura et al. 1998; Mark et al. 2020; Pittsenbarger et al. 2017; Schultz et al. 2015; Wang et al. 2010). Nevertheless, given the high rates of incarceration and contact with the law enforcement in the US (Asad & Clair, 2018; Kaeble, 2020; The Sentencing Project, 2022), the occurrence of legal problems among more general patient populations should be not negligible. The estimates in the general population samples bear out that legal problems are somewhat common (Additional file 2).

### Limitations

The study is limited in that it did not specifically include civil legal problems like unsafe housing, unfair employment, or family law (Currie, 2009; Sandel et al. 2010). However, some articles may have included these issues and if we had included these issues, we would have likely seen even more variation. In addition, we limited our studies to those in healthcare-related settings. Other studies have looked at population level percentages or measurement in community settings, thus they may have had different foci and resulted in different strategies.

## Conclusions

Increasingly healthcare organizations are screening patients for various social risk factors to drive referral decisions and support community needs assessments. The operationalization of legal problems in measurement approaches is variable often without strong evidence of construct validity.

### Supplementary Information

**Additional file 1.** 

**Additional file 2.**

## Data Availability

Data sharing not applicable to this article as no datasets were generated or analysed during the current study.
