# Supplementary material for: How legal problems are conceptualized and measured in healthcare settings: a systematic review
Source: Health Justice. 2023 Nov 18;11:48. doi: 10.1186/s40352-023-00246-5 (PMC10656991; doi:10.1186/s40352-023-00246-5)
Supplement: Supplementary file 1 — Additional file 1. [file 40352_2023_246_MOESM1_ESM.docx]

**Appendix A.**

**Search Strategy for Legal Status Screening in Healthcare**

**Ovid MEDLINE(R) and Epub Ahead of Print, In-Process, In-Data-Review & Other Non-Indexed Citations and Daily**

(((Prisoners/ or Criminals/ or Juvenile Delinquency/ OR (((legal or justice) adj2 (involve* or criminal or system)) or (arrest* adj2 (record* OR history* OR police)) or incarceration OR incarcerated OR jail OR jails OR prison* OR (corrections adj3 (health OR service* OR criminal* OR care OR system*)) OR offender* OR correctional OR parole OR probation OR community-supervision OR structural-vulnerabilit* OR ((criminal OR crime) adj2 record)).ab,ti.) NOT cardiac-arrest*.ab,ti) OR "Social Determinants of Health"/ OR (determinant adj2 (health OR structural)).ti,ab.)

AND

(Mass screening/ or electronic health records/ or self report/ OR (history OR self-report* OR (electronic adj2 record*) OR medical-record* OR screening or checklist* OR screened OR linkage OR ((adult* OR adolescent* OR teenager* OR patient* OR individual* OR people) adj3 (survey* OR ask* OR question*)) OR ((tool* OR instrument* OR standard*) adj2 (survey* OR assessment*))).ti,ab.)

AND

(primary care/ OR emergency service, hospital/ OR exp ambulatory care facilities/ OR (primary-care OR (emergency adj2 (ward* OR room* OR department* OR service OR care OR unit)) OR ambulatory-care OR urgent-care OR clinic OR clinics OR health-care OR healthcare OR health-services OR health-center* OR treatment-center* OR rehabilitation-center* OR family-planning-center* OR surgicenter* OR health-system).ti,ab.)

NOT ((exp africa/ or exp asia/ or exp europe/ or exp islands/ or exp oceania/ or exp canada/ or exp mexico/ or exp South America/ or exp Central America/) not ((exp africa/ or exp asia/ or exp europe/ or exp islands/ or exp oceania/ or exp canada/ or exp mexico/ or exp South America/ or exp Central America/) and (exp United States/ or African americans/ or Indians, North American/ or Asian americans/ or Hispanic americans/ or America*.mp. or united states.mp. OR refugee*)))

NOT (editorial OR news OR letter OR comment).pt

Limits: English

Results: 1793

**PsycINFO via EBSCO**

(DE "Prisoners" OR DE "Criminal Offenders" OR DE "Female Criminal Offenders" OR DE "Male Criminal Offenders" OR DE "Mentally Ill Offenders" OR DE "Criminal Record" OR DE "Incarceration" OR DE "Juvenile Delinquency" OR DE "Female Delinquency" OR DE "Male Delinquency" OR TI(((legal or justice) NEAR/2 (involve* or criminal or system)) or (arrest* NEAR/2 (record* OR history* OR police)) or incarceration OR incarcerated OR jail OR jails OR prison* OR (corrections NEAR/3 (health OR service* OR criminal* OR care OR system*)) OR (determinant NEAR/2 (health OR structural)) OR correctional OR parole OR probation OR community-supervision OR structural-vulnerabilit* OR ((criminal OR crime) NEAR/2 record) NOT cardiac-arrest*) OR AB(((legal or justice) NEAR/2 (involve* or criminal or system)) or (arrest* NEAR/2 (record* OR history* OR police)) or incarceration OR incarcerated OR jail OR jails OR prison* OR (corrections NEAR/3 (health OR service* OR criminal* OR care OR system*)) OR (determinant NEAR/2 (health OR structural)) OR correctional OR parole OR probation OR community-supervision OR structural-vulnerabilit* OR ((criminal OR crime) NEAR/2 record) NOT cardiac-arrest*))

AND

(DE "Health Screening" OR DE "Screening" OR DE "Screening Tests" OR DE "Medical Records" OR DE "Client Records" OR DE "Electronic Health Records" OR DE "Self-Report" OR TI (history OR self-report* OR (electronic NEAR/2 record*) OR medical-record* OR screening or checklist* OR screened OR linkage OR ((adult* OR adolescent* OR teenager* OR patient* OR individual* OR people) NEAR/3 (survey* OR ask* OR question*)) OR ((tool* OR instrument* OR standard*) NEAR/2 (survey* OR assessment*))) OR AB (history OR self-report* OR (electronic NEAR/2 record*) OR medical-record* OR screening or checklist* OR screened OR linkage OR ((adult* OR adolescent* OR teenager* OR patient* OR individual* OR people) NEAR/3 (survey* OR ask* OR question*)) OR ((tool* OR instrument* OR standard*) NEAR/2 (survey* OR assessment*))))

AND

(DE "Behavioral Health Services" OR DE "Primary Health Care" OR DE "Outpatient Treatment" OR DE "Emergency Services" OR DE "Clinics" OR DE "Psychiatric Clinics" OR DE "Walk In Clinics" OR DE "Community Mental Health Centers" OR TI (primary-care OR (emergency NEAR/2 (ward* OR room* OR department* OR service OR care OR unit)) OR ambulatory-care OR urgent-care OR clinic OR clinics OR health-care OR healthcare OR health-services OR health-center* OR treatment-center* OR rehabilitation-center* OR family-planning-center* OR surgicenter* OR health-system) OR AB (primary-care OR (emergency NEAR/2 (ward* OR room* OR department* OR service OR care OR unit)) OR ambulatory-care OR urgent-care OR clinic OR clinics OR health-care OR healthcare OR health-services OR health-center* OR treatment-center* OR rehabilitation-center* OR family-planning-center* OR surgicenter* OR health-system))

NOT

(((ZY "afghanistan") or (ZY "africa") or (ZY "albania") or (ZY "algeria") or (ZY "american samoa") or (ZY "andorra") or (ZY "angola") or (ZY "anguilla") or (ZY "antarctica") or (ZY "antigua and barbuda") or (ZY "arctic regions") or (ZY "argentina") or (ZY "armenia") or (ZY "aruba") or (ZY "ashmore and cartier islands") or (ZY "asia") or (ZY "australia") or (ZY "austria") or (ZY "azerbaijan") or (ZY "bahamas") or (ZY "bahrain") or (ZY "baker island") or (ZY "balkan states") or (ZY "baltic states") or (ZY "bangladesh") or (ZY "barbados") or (ZY "belarus") or (ZY "belgium") or (ZY "belize") or (ZY "benin") or (ZY "bermuda") or (ZY "bhutan") or (ZY "bolivia") or (ZY "bosnia-herzegovina") or (ZY "botswana") or (ZY "bouvet island") or (ZY "brazil") or (ZY "british virgin islands") or (ZY "brunei") or (ZY "bulgaria") or (ZY "burkina faso") or (ZY "burundi") or (ZY "cambodia") or (ZY "cameroon") or (ZY "canada") or (ZY "cape verde") or (ZY "cape verde islands") or (ZY "caribbean") or (ZY "cayman islands") or (ZY "central african republic") or (ZY "central america") or (ZY "chad") or (ZY "channel islands") or (ZY "chile") or (ZY "china") or (ZY "christmas island") or (ZY "cocos (keeling) islands") or (ZY "colombia") or (ZY "commonwealth of independent states") or (ZY "comoros") or (ZY "cook islands") or (ZY "coral sea islands") or (ZY "costa rica") or (ZY "croatia") or (ZY "cuba") or (ZY "curacao") or (ZY "cyprus") or (ZY "czech republic") or (ZY "czechoslovakia") or (ZY "democratic people's republic of korea") or (ZY "democratic republic of congo") or (ZY "denmark") or (ZY "djibouti") or (ZY "dominica") or (ZY "dominican republic") or (ZY "east timor") or (ZY "eastern europe") or (ZY "ecuador") or (ZY "egypt") or (ZY "el salvador") or (ZY "england") or (ZY "equatorial guinea") or (ZY "eritrea") or (ZY "estonia") or (ZY "ethiopia") or (ZY "europa island") or (ZY "europe") or (ZY "falkland islands") or (ZY "faroe islands") or (ZY "fiji") or (ZY "finland") or (ZY "france") or (ZY "french guiana") or (ZY "french polynesia") or (ZY "french southern and antarctic lands") or (ZY "gabon") or (ZY "gambia") or (ZY "gaza strip") or (ZY "georgia") or (ZY "germany") or (ZY "ghana") or (ZY "gibraltar") or (ZY "glorioso islands") or (ZY "great britain") or (ZY "greece") or (ZY "greenland") or (ZY "grenada") or (ZY "guadeloupe") or (ZY "guam") or (ZY "guatemala") or (ZY "guernsey") or (ZY "guinea") or (ZY "guinea-bissau") or (ZY "guyana") or (ZY "haiti") or (ZY "heard island and mcdonald islands") or (ZY "holy see (vatican city)") or (ZY "honduras") or (ZY "hong kong") or (ZY "howland island") or (ZY "hungary") or (ZY "iceland") or (ZY "india") or (ZY "indonesia") or (ZY "iran") or (ZY "iraq") or (ZY "ireland") or (ZY "israel") or (ZY "italy") or (ZY "ivory coast") or (ZY "jamaica") or (ZY "jan mayen") or (ZY "japan") or (ZY "jarvis island") or (ZY "jersey") or (ZY "johnston atoll") or (ZY "jordan") or (ZY "juan de nova island") or (ZY "kazakhstan") or (ZY "kenya") or (ZY "kiribati") or (ZY "korea") or (ZY "kosovo") or (ZY "kuwait") or (ZY "kyrgyzstan") or (ZY "laos") or (ZY "latin america") or (ZY "latvia") or (ZY "lebanon") or (ZY "lesotho") or (ZY "liberia") or (ZY "libya") or (ZY "liechtenstein") or (ZY "lithuania") or (ZY "luxembourg") or (ZY "macao") or (ZY "macau") or (ZY "macedonia") or (ZY "madagascar") or (ZY "malawi") or (ZY "malaysia") or (ZY "maldives") or (ZY "mali") or (ZY "malta") or (ZY "man, isle of") or (ZY "marshall islands") or (ZY "martinique") or (ZY "mauritania") or (ZY "mauritius") or (ZY "mayotte") or (ZY "mexico") or (ZY "micronesia (federated states of)") or (ZY "middle east") or (ZY "midway islands") or (ZY "moldova") or (ZY "monaco") or (ZY "mongolia") or (ZY "montenegro") or (ZY "montserrat") or (ZY "morocco") or (ZY "mozambique") or (ZY "myanmar") or (ZY "namibia") or (ZY "nauru") or (ZY "navasa island") or (ZY "nepal") or (ZY "netherlands") or (ZY "netherlands antilles") or (ZY "new caledonia") or (ZY "new zealand") or (ZY "nicaragua") or (ZY "niger") or (ZY "nigeria") or (ZY "niue") or (ZY "norfolk island") or (ZY "north vietnam") or (ZY "northern ireland") or (ZY "northern mariana islands") or (ZY "norway") or (ZY "oceania") or (ZY "oman") or (ZY "pacific islands") or (ZY "pakistan") or (ZY "palau") or (ZY "palestine") or (ZY "palmyra atoll") or (ZY "panama") or (ZY "papua new guinea") or (ZY "paraguay") or (ZY "peru") or (ZY "philippines") or (ZY "pitcairn islands") or (ZY "poland") or (ZY "portugal") or (ZY "puerto rico") or (ZY "qatar") or (ZY "republic of congo") or (ZY "republic of korea") or (ZY "republic of serbia") or (ZY "reunion") or (ZY "romania") or (ZY "russia") or (ZY "rwanda") or (ZY "samoa") or (ZY "san marino") or (ZY "sao tome and principe") or (ZY "saudi arabia") or (ZY "scandinavia") or (ZY "scotland") or (ZY "senegal") or (ZY "serbia and montenegro") or (ZY "seychelles") or (ZY "sierra leone") or (ZY "singapore") or (ZY "slovak republic") or (ZY "slovakia") or (ZY "slovenia") or (ZY "solomon islands") or (ZY "somalia") or (ZY "south africa") or (ZY "south america") or (ZY "south sudan") or (ZY "spain") or (ZY "spratly islands") or (ZY "sri lanka") or (ZY "st helena") or (ZY "st kitts") or (ZY "st kitts and nevis") or (ZY "st lucia") or (ZY "st pierre and miquelon") or (ZY "st vincent and the grenadines") or (ZY "sudan") or (ZY "surinam") or (ZY "suriname") or (ZY "svalbard") or (ZY "swaziland") or (ZY "sweden") or (ZY "switzerland") or (ZY "syria") or (ZY "taiwan") or (ZY "tajikistan") or (ZY "tanzania") or (ZY "thailand") or (ZY "tibet") or (ZY "togo") or (ZY "tokelau") or (ZY "tonga") or (ZY "trinidad and tobago") or (ZY "tunisia") or (ZY "turkey") or (ZY "turkmenistan") or (ZY "turks and caicos islands") or (ZY "tuvalu") or (ZY "uganda") or (ZY "ukraine") or (ZY "united arab emirates") or (ZY "united kingdom") or (ZY "uruguay") or (ZY "ussr") or (ZY "uzbekistan") or (ZY "vanuatu") or (ZY "venezuela") or (ZY "vietnam") or (ZY "wake island") or (ZY "wales") or (ZY "wallis and futuna") or (ZY "west bank") or (ZY "west indies") or (ZY "western europe") or (ZY "western sahara") or (ZY "yemen") or (ZY "yugoslavia") or (ZY "zambia") or (ZY "zimbabwe")) NOT (ZY "us"))

Limits: Peer reviewed journals; English

Results: 665

**CINAHL Complete (EBSCO)**

(MH "Prisoners" OR MH "Public Offenders" OR MH "Juvenile Offenders" OR MH "Mentally Ill Offenders" OR MH "Repeat Offenders" OR MH "Sex Offenders" OR MH "Juvenile Delinquency" OR TI(((legal or justice) NEAR/2 (involve* or criminal or system)) or (arrest* NEAR/2 (record* OR history* OR police)) or incarceration OR incarcerated OR jail OR jails OR prison* OR (corrections NEAR/3 (health OR service* OR criminal* OR care OR system*)) OR (determinant NEAR/2 (health OR structural)) OR correctional OR parole OR probation OR community-supervision OR structural-vulnerabilit* OR ((criminal OR crime) NEAR/2 record) NOT cardiac-arrest*) OR AB(((legal or justice) NEAR/2 (involve* or criminal or system)) or (arrest* NEAR/2 (record* OR history* OR police)) or incarceration OR incarcerated OR jail OR jails OR prison* OR (corrections NEAR/3 (health OR service* OR criminal* OR care OR system*)) OR (determinant NEAR/2 (health OR structural)) OR correctional OR parole OR probation OR community-supervision OR structural-vulnerabilit* OR ((criminal OR crime) NEAR/2 record) NOT cardiac-arrest*))

AND

(MH "Health Screening" OR MH "Patient History Taking" OR MH "Self Report" OR MH "Medical Records" OR MH "Electronic Health Records" OR TI (history OR self-report* OR (electronic NEAR/2 record*) OR medical-record* OR screening or checklist* OR screened OR linkage OR ((adult* OR adolescent* OR teenager* OR patient* OR individual* OR people) NEAR/3 (survey* OR ask* OR question*)) OR ((tool* OR instrument* OR standard*) NEAR/2 (survey* OR assessment*))) OR AB (history OR self-report* OR (electronic NEAR/2 record*) OR medical-record* OR screening or checklist* OR screened OR linkage OR ((adult* OR adolescent* OR teenager* OR patient* OR individual* OR people) NEAR/3 (survey* OR ask* OR question*)) OR ((tool* OR instrument* OR standard*) NEAR/2 (survey* OR assessment*))))

AND

(MH "Primary Health Care" OR MH "Emergency Service+" OR MH "Ambulatory Care Facilities" OR MH "Ambulatory Care" OR MH "Community Health Centers" OR TI (primary-care OR (emergency NEAR/2 (ward* OR room* OR department* OR service OR care OR unit)) OR ambulatory-care OR urgent-care OR clinic OR clinics OR health-care OR healthcare OR health-services OR health-center* OR treatment-center* OR rehabilitation-center* OR family-planning-center* OR surgicenter* OR health-system) OR AB (primary-care OR (emergency NEAR/2 (ward* OR room* OR department* OR service OR care OR unit)) OR ambulatory-care OR urgent-care OR clinic OR clinics OR health-care OR healthcare OR health-services OR health-center* OR treatment-center* OR rehabilitation-center* OR family-planning-center* OR surgicenter* OR health-system))

NOT ((MH "Africa+" OR MH "Antarctic Regions" OR MH "Arctic Regions" OR MH "Asia+" OR MH "Atlantic Islands+" OR MH "Australia+" OR MH "Europe+" OR MH "Historical Geographic Locations+" OR MH "Indian Ocean Islands+" OR MH "Low and Middle Income Countries" OR MH "Pacific Islands+" OR MH "Central America+" OR MH "Latin America" OR MH "Canada+" OR MH "Greenland" OR MH "Mexico" OR MH "South America+" OR MH "West Indies+") NOT ((MH "Africa+" OR MH "Antarctic Regions" OR MH "Arctic Regions" OR MH "Asia+" OR MH "Atlantic Islands+" OR MH "Australia+" OR MH "Europe+" OR MH "Historical Geographic Locations+" OR MH "Indian Ocean Islands+" OR MH "Low and Middle Income Countries" OR MH "Pacific Islands+" OR MH "Central America+" OR MH "Latin America" OR MH "Canada+" OR MH "Greenland" OR MH "Mexico" OR MH "South America+" OR MH "West Indies+") AND (MH "United States+" OR TI (America* OR united states) OR AB (America* OR united states) OR refugee*)))

Limits: English, Academic Journals

Results: 677

**Criminal Justice Index**

(MAINSUBJECT.EXACT("Prisoners") OR MAINSUBJECT.EXACT("Imprisonment") OR MAINSUBJECT.EXACT("Criminals") OR MAINSUBJECT.EXACT("Habitual offenders") OR MAINSUBJECT.EXACT("Juvenile offenders") OR MAINSUBJECT.EXACT("Juvenile delinquency") OR TIAB(((legal or justice) NEAR/2 (involve* or criminal or system)) or (arrest* NEAR/2 (record* OR history* OR police)) or incarceration OR incarcerated OR jail OR jails OR prison* OR (corrections NEAR/3 (health OR service* OR criminal* OR care OR system*)) OR (determinant NEAR/2 (health OR structural)) OR correctional OR parole OR probation OR community-supervision OR structural-vulnerabilit* OR ((criminal OR crime) NEAR/2 record) NOT cardiac-arrest*))

AND

(MAINSUBJECT.EXACT("Medical screening") OR MAINSUBJECT.EXACT("Medical records") OR MAINSUBJECT.EXACT("Electronic health records") OR TIAB(history OR self-report* OR (electronic NEAR/2 record*) OR medical-record* OR screening or checklist* OR screened OR linkage OR ((adult* OR adolescent* OR teenager* OR patient* OR individual* OR people) NEAR/3 (survey* OR ask* OR question*)) OR ((tool* OR instrument* OR standard*) NEAR/2 (survey* OR assessment*))))

AND

(MAINSUBJECT.EXACT("Community health care") OR MAINSUBJECT.EXACT("Primary care") OR MAINSUBJECT.EXACT("Ambulatory care") OR MAINSUBJECT.EXACT("Emergency services") OR MAINSUBJECT.EXACT("Clinics") OR TIAB(primary-care OR (emergency NEAR/2 (ward* OR room* OR department* OR service OR care OR unit)) OR ambulatory-care OR urgent-care OR clinic OR clinics OR health-care OR healthcare OR health-services OR health-center* OR treatment-center* OR rehabilitation-center* OR family-planning-center* OR surgicenter* OR health-system) OR AB (primary-care OR (emergency NEAR/2 (ward* OR room* OR department* OR service OR care OR unit)) OR ambulatory-care OR urgent-care OR clinic OR clinics OR health-care OR healthcare OR health-services OR health-center* OR treatment-center* OR rehabilitation-center* OR family-planning-center* OR surgicenter* OR health-system))

Limits: English; academic journal

Excluded: all articles with non-US locations

Results: 375

**Google Scholar**

Incarceration|criminals|prisoners|arrested screening|health-record|checklist|surveyed|questionnaire|tool|instrument primary|ambulatory|emergency|clinic|center reentry|"recently released"|Determinants

Downloaded 100 results
